# Clinical characteristics and outcomes of children with single or co-detected rhinovirus-associated acute respiratory infection in Middle Tennessee

**DOI:** 10.1186/s12879-023-08084-4

**Published:** 2023-03-07

**Authors:** Justin Z. Amarin, Molly Potter, Jyotsna Thota, Danielle A. Rankin, Varvara Probst, Zaid Haddadin, Laura S. Stewart, Ahmad Yanis, Rana Talj, Herdi Rahman, Tiffanie M. Markus, James Chappell, Mary Lou Lindegren, William Schaffner, Andrew J. Spieker, Natasha B. Halasa

**Affiliations:** 1 Division of Pediatric Infectious Diseases, Department of Pediatrics, Vanderbilt University Medical Center, 1161 21st Avenue South, Medical Center North D7235, Nashville, TN 37232 USA; 2 Vanderbilt Epidemiology PhD Program, Vanderbilt University School of Medicine, Nashville, TN USA; 3 Department of Health Policy, Vanderbilt University Medical Center, Nashville, TN USA; 4 Department of Medicine, Vanderbilt University Medical Center, Nashville, TN USA; 5 Department of Biostatistics, Vanderbilt University Medical Center, Nashville, TN USA

**Keywords:** Rhinovirus, Common cold, Coinfection, Epidemiology, Tennessee

## Abstract

**Background:**

Rhinovirus (RV) is one of the most common etiologic agents of acute respiratory infection (ARI), which is a leading cause of morbidity and mortality in young children. The clinical significance of RV co-detection with other respiratory viruses, including respiratory syncytial virus (RSV), remains unclear. We aimed to compare the clinical characteristics and outcomes of children with ARI-associated RV-only detection and those with RV co-detection—with an emphasis on RV/RSV co-detection.

**Methods:**

We conducted a prospective viral surveillance study (11/2015–7/2016) in Nashville, Tennessee. Children < 18 years old who presented to the emergency department (ED) or were hospitalized with fever and/or respiratory symptoms of < 14 days duration were eligible if they resided in one of nine counties in Middle Tennessee. Demographics and clinical characteristics were collected by parental interviews and medical chart abstractions. Nasal and/or throat specimens were collected and tested for RV, RSV, metapneumovirus, adenovirus, parainfluenza 1–4, and influenza A–C using reverse transcription quantitative polymerase chain reaction assays. We compared the clinical characteristics and outcomes of children with RV-only detection and those with RV co-detection using Pearson’s *χ*^2^ test for categorical variables and the two-sample *t*-test with unequal variances for continuous variables.

**Results:**

Of 1250 children, 904 (72.3%) were virus-positive. RV was the most common virus (*n* = 406; 44.9%), followed by RSV (*n* = 207; 19.3%). Of 406 children with RV, 289 (71.2%) had RV-only detection, and 117 (28.8%) had RV co-detection. The most common virus co-detected with RV was RSV (*n* = 43; 36.8%). Children with RV co-detection were less likely than those with RV-only detection to be diagnosed with asthma or reactive airway disease both in the ED and in-hospital. We did not identify differences in hospitalization, intensive care unit admission, supplemental oxygen use, or length of stay between children with RV-only detection and those with RV co-detection.

**Conclusion:**

We found no evidence that RV co-detection was associated with poorer outcomes. However, the clinical significance of RV co-detection is heterogeneous and varies by virus pair and age group. Future studies of RV co-detection should incorporate analyses of RV/non-RV pairs and include age as a key covariate of RV contribution to clinical manifestations and infection outcomes.

**Supplementary Information:**

The online version contains supplementary material available at 10.1186/s12879-023-08084-4.

## Background

Acute respiratory infection (ARI) is a leading cause of morbidity and mortality in young children and accounts for 20–40% of hospitalizations in this age group [1, 2]. The most common etiologic agents of ARI are viral pathogens, among which rhinovirus (RV) is a leading cause. Though usually associated with upper respiratory illness, RV is also associated with lower respiratory illnesses such as asthma exacerbations, bronchiolitis, and pneumonia [3].

Due to the increased availability and use of multipathogen molecular testing in clinical settings, RV is commonly detected alongside multiple respiratory viruses, including respiratory syncytial virus (RSV) [4–6]. The prevalence of viral co-detection in children with ARI generally ranges from 10 to 30% and is higher in those who are hospitalized, younger, and attend day care [7–9]. However, it remains unclear whether co-detections are associated with more severe illness [8]. Some studies have reported worse outcomes in children with viral co-detection [10], while others have reported no differences [7, 9, 11, 12]. In addition, the use of aggregated data assumes that the relationship between viral co-detection and disease severity is homogeneous across virus pairs. Therefore, analyses of aggregated data may mask the clinical significance of specific pairs [8, 13]. Therefore, we aimed to compare the clinical characteristics and outcomes of children with ARI-associated RV-only detection and those with RV co-detection—with an emphasis on RV/RSV co-detection.

## Methods

### Study design

We conducted a prospective viral surveillance study from November 15, 2015, to July 15, 2016, at Monroe Carell Jr. Children’s Hospital at Vanderbilt in Nashville, Tennessee. Children were enrolled 5 days per week (Monday, Wednesday, Thursday, Friday, and Sunday) from both the emergency department (ED) and inpatient service. Patients admitted to the ED were actively screened for symptoms of ARI.

### Study setting and population

Children < 18 years old who presented with fever and/or respiratory symptoms of duration < 14 days were eligible if they resided in the study catchment area, which included the following nine counties in Middle Tennessee: Cheatham, Davidson, Dickson, Montgomery, Robertson, Rutherford, Sumner, Williamson, and Wilson. We selected the counties based on geographic proximity to Davidson County. We excluded children who were previously enrolled for the same episode of ARI in the past week, newborns who were never discharged, children with fever and neutropenia or a known nonrespiratory cause of symptoms, and children who were hospitalized for > 48 h.

### Data collection

Research staff interviewed parents/guardians and collected data using a standardized case report form. They then performed medical chart abstractions to record additional information on clinical characteristics (e.g., final clinical diagnosis) and outcomes, including hospitalization, intensive care unit (ICU) admission, supplemental oxygen use, and length of stay. Any child readmitted more than a week after their initial enrollment with fever and/or respiratory symptoms of duration < 14 days was considered a new and unique case.

### Sample collection and testing

After obtaining consent, research staff collected nose and/or throat swabs and combined them in a viral transport medium (BD) if both were collected. Specimens were stored at 2–8 °C, transported to the laboratory, and divided into multiple aliquots. Total nucleic acid extraction was performed using the Roche MagNA Pure LC automated extraction system. Using reverse transcription quantitative polymerase chain reaction (RT-qPCR) assays, all specimens were tested for rhinovirus (RV)/enterovirus, RSV, adenovirus (AdV), influenza (Flu) A, B, and C, metapneumovirus (MPV), parainfluenza virus (PIV)-1–4, and human RNase P (as an indicator of specimen quality). Cycle threshold (Ct) values served as a surrogate for viral load. Specimens were considered positive if the Ct value was less than 45 cycles. All laboratory results and clinical data were entered into a REDCap database [14]. We did not include the results of clinical viral testing in our study.

### Statistical analysis

We determined descriptive statistics as absolute/relative frequency, mean/standard deviation (SD), or median/interquartile range (IQR) as appropriate. We used Pearson’s *χ*^2^ test and the two-sample *t*-test with unequal variances to compare categorical and continuous variables, respectively, between children with RV-only detection and those with RV co-detection. To account for children who were enrolled more than once and adjust for important covariates, we used generalized estimating equations with a logistic link and a working independence correlation structure to estimate adjusted odds ratios (aORs) and 95% confidence intervals (95% CIs) comparing the odds of hospitalization between children with RV-only detection and those with RV co-detection. We included in the model age, sex, race and Hispanic origin, tobacco smoke exposure, and history of asthma as covariates [15]. Finally, we performed a subgroup analysis of children < 2 years old with RV/RSV co-detection, RV-only detection, or RSV-only detection. Significance was determined to be achieved at a nominal level of α = 0.05 (two-tailed, where appropriate). All analyses were conducted using R (version 4.1.2).

### Ethical considerations

The study protocol was approved by the Vanderbilt University Institutional Review Board, and written informed consent was obtained from parents/guardians.

## Results

### Study population

We screened 2300 children for eligibility and enrolled 1255 (54.6%); 1184 (97.3%) were enrolled once, and 33 (2.7%) more than once. Of children enrolled more than once, one was enrolled five times, another two were enrolled three times, and 30 were enrolled twice. Of those enrolled, we collected nose and/or throat swabs from 1251 children (99.7%) and subsequently excluded one child (0.1%) with an inconclusive test result for RV. The median age of the cohort was 2.2 years (IQR, 0.8–5.5 years). Of the 1,250 children we included in our analysis, 904 (72.3%) were virus-positive. A nasal swab alone was collected in one case (0.1%), a throat swab alone in another (0.1%), and both in the remaining 1,248 cases (99.8%). RV was the most common virus (*n* = 406; 44.9%), followed by RSV (*n* = 207; 19.3%), AdV (*n* = 152; 14.2%), Flu (*n* = 150; 14.0%), MPV (*n* = 106; 9.9%), and PIV (*n* = 51; 4.8%). Additional file 1: Table S1 presents comparisons of clinical characteristics and outcomes between children who were RV-positive and those who were RV-negative but positive for another respiratory virus.

### RV co-detection

**Fig. 1:**
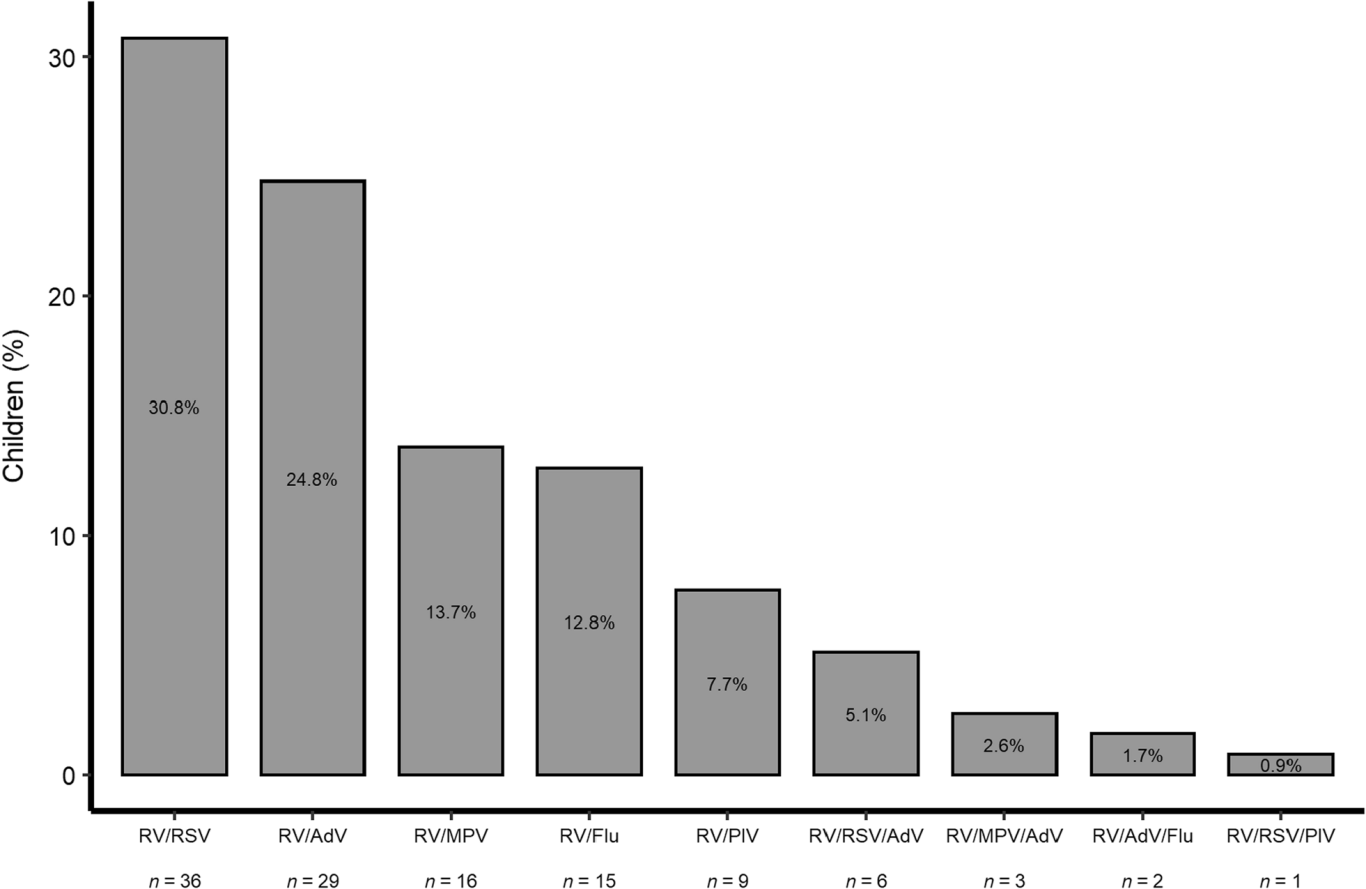
Respiratory viruses co-detected with RV in 117 children with acute respiratory infection in Middle Tennessee. *RV* rhinovirus, *RSV* respiratory syncytial virus, *AdV* adenovirus, *MPV* metapneumovirus, *Flu* influenza, *PIV* parainfluenza virus

Of 406 children with RV, 289 (71.2%) had RV-only detection and 117 (28.8%) had RV co-detection (Fig. 1). Four children were enrolled twice (i.e., *n* = 402 unique children). The most common virus co-detected with RV was RSV (*n* = 43; 36.8%), followed by AdV (*n* = 40; 34.2%), MPV (*n* = 19; 16.2%), Flu (*n* = 17; 14.5%), and PIV (*n* = 10; 8.5%). In 105 cases (89.7%), one other virus was co-detected with RV, and in 12 cases (10.3%), two other viruses were co-detected with RV. The most frequently detected virus pair was RV/RSV (*n* = 36; 34.3% of all pairs) and the most frequently detected virus triplet was RV/RSV/AdV (*n* = 6; 50.0% of all triplets). The mean Ct value for RV was higher in children with RV co-detection than in children with RV-only detection (32.3 ± 4.5 vs. 28.2 ± 5.7; *p* < 0.001). Similarly, the mean Ct value for RV was higher in children with RV/RSV co-detection than in children with RV-only detection (32.0 ± 4.6 vs. 28.2 ± 5.7; *p* < 0.001). The mean Ct value for RSV was also higher in children with RV/RSV co-detection than in children with RSV-only detection (28.7 ± 5.8 vs. 26.4 ± 4.6; *p* = 0.029).

### Clinical characteristics of children with RV-only detection and RV co-detection

The mean age at enrollment of the 406 children with RV was 3.8 years (SD, 4.1 years); 189 (46.6%) were < 2 years old, 100 (24.6%) were 2–4 years old, 79 (19.5%) were 5–9 years old, and 38 (9.4%) were 10–17 years old. Boys outnumbered girls 247 (60.8%) to 159 (39.2%) for a male-to-female ratio of 1.6. By race and Hispanic origin, 158 children (38.9%) were non-Hispanic black, 123 (30.3%) were non-Hispanic white, 97 (23.9%) were Hispanic, and 28 (6.9%) were non-Hispanic other.

The clinical characteristics of children with RV-only detection and those with RV co-detection are compared in Table 1. Children with RV co-detection were, on average, younger, had a longer mean duration of illness at presentation, were more likely to attend day care or preschool, were more likely to have received antibiotics for the illness before presentation, and were less likely to have a history of asthma than children with RV-only detection. Children with RV/RSV co-detection were older, had a longer duration of illness at presentation, were more likely to have received antibiotics for the illness before presentation, and were less likely to have a history of asthma than children with RV-only detection. Race and Hispanic origin was associated with RV/RSV co-detection (*p* = 0.042), with non-Hispanic white children being the most likely to have RV/RSV co-detection.

**Table 1:** Clinical characteristics and outcomes of 406 children with RV-only detection or RV co-detection in Middle Tennessee

Clinical characteristics	RV-only detection (*n* = 289)	RV co-detection (*n* = 117)	*p* value*	RV/RSV co-detection (*n* = 36)	*p* value*
Age at enrollment in years					
Mean (SD)	4.3 (4.4)	2.6 (2.7)	**< 0.001**	1.1 (1.2)	**< 0.001**
Median (IQR)	2.8 (0.8–6.3)	1.5 (0.8–3.4)		0.6 (0.2–1.7)	
Male—*n* (%)	174 (60.2)	73 (62.4)	0.68	19 (52.8)	0.39
Race and Hispanic origin—*n* (%)			0.63		**0.042**
Hispanic	70 (24.2)	27 (23.1)		7 (19.4)	
Non-Hispanic white	87 (30.1)	36 (30.8)		14 (38.9)	
Non-Hispanic black	115 (39.8)	43 (36.8)		9 (25.0)	
Non-Hispanic other	17 (5.9)	11 (9.4)		6 (16.7)	
Illness duration at presentation in days					
Mean (SD)	3.3 (2.2)	4.1 (2.7)	**0.004**	4.9 (2.9)	**0.001**
Median (IQR)	3 (2–4)	3 (2–6)		4.5 (3–7)	
Breastfeeding history^a^—*n* (%)	90/123 (73.2)	45/66 (68.2)	0.47	19/28	0.57
Premature birth^a^—*n* (%)	22/121 (18.2)	14/64 (21.9)	0.55	6/26 (23.1)	0.56
Day care or preschool attendance^b^—*n* (%)	52/190 (27.4)	38/94 (40.4)	**0.026**	12/35 (34.3)	0.40
School attendance^c^—*n* (%)	87/94 (92.6)	22/23 (95.7)	0.60	1/1 (100.0)	0.78
Tobacco smoke exposure—*n* (%)	111 (38.4)	47/116 (40.5)	0.69	17/35 (48.6)	0.25
Gestational tobacco smoke exposure^a^—*n* (%)	44/285 (15.4)	15/116 (12.9)	0.52	8/35 (22.9)	0.26
Prior antiviral use—*n* (%)	1/287 (0.3)	0/116	0.52	0/35	0.73
Prior antibiotic use—*n* (%)	12/286 (4.2)	16 (13.7)	**< 0.001**	7/36 (19.4)	**< 0.001**
Underlying medical condition—*n* (%)	161 (55.7)	54 (46.2)	0.081	15 (41.7)	0.11
History of asthma—*n* (%)	99 (34.3)	15 (12.8)	**< 0.001**	2 (5.6)	**< 0.001**
Outcomes					
Hospitalized—*n* (%)	95 (32.9)	28 (23.9)	0.076	16 (44.4)	0.17
ICU admission—*n* (%)	9 (9.5)	4 (14.3)	0.47	3 (18.8)	0.27
Supplemental oxygen use—*n* (%)	37 (38.9)	16 (57.1)	0.088	10 (62.5)	0.078
Length of stay—days					
Mean (SD)	2.0 (2.6)	2.9 (2.2)	0.097	3.2 (2.5)	0.084
Median (IQR)	1 (1–2)	2 (1.75–4)		2 (1.75–4.25)	

The most common RV co-detection pair (RV/RSV) is presented separately

Values in bold indicate *p* < 0.05

^a^Children < 2 years old.

^b^Children < 5 years old.

^c^Children ≥ 5 years old

*p values were calculated using Pearson’s *χ*^2^ test for categorical variables and the two-sample *t*-test with unequal variances for continuous variables

### Clinical presentation of children with RV-only detection and RV co-detection

**Fig. 2:**
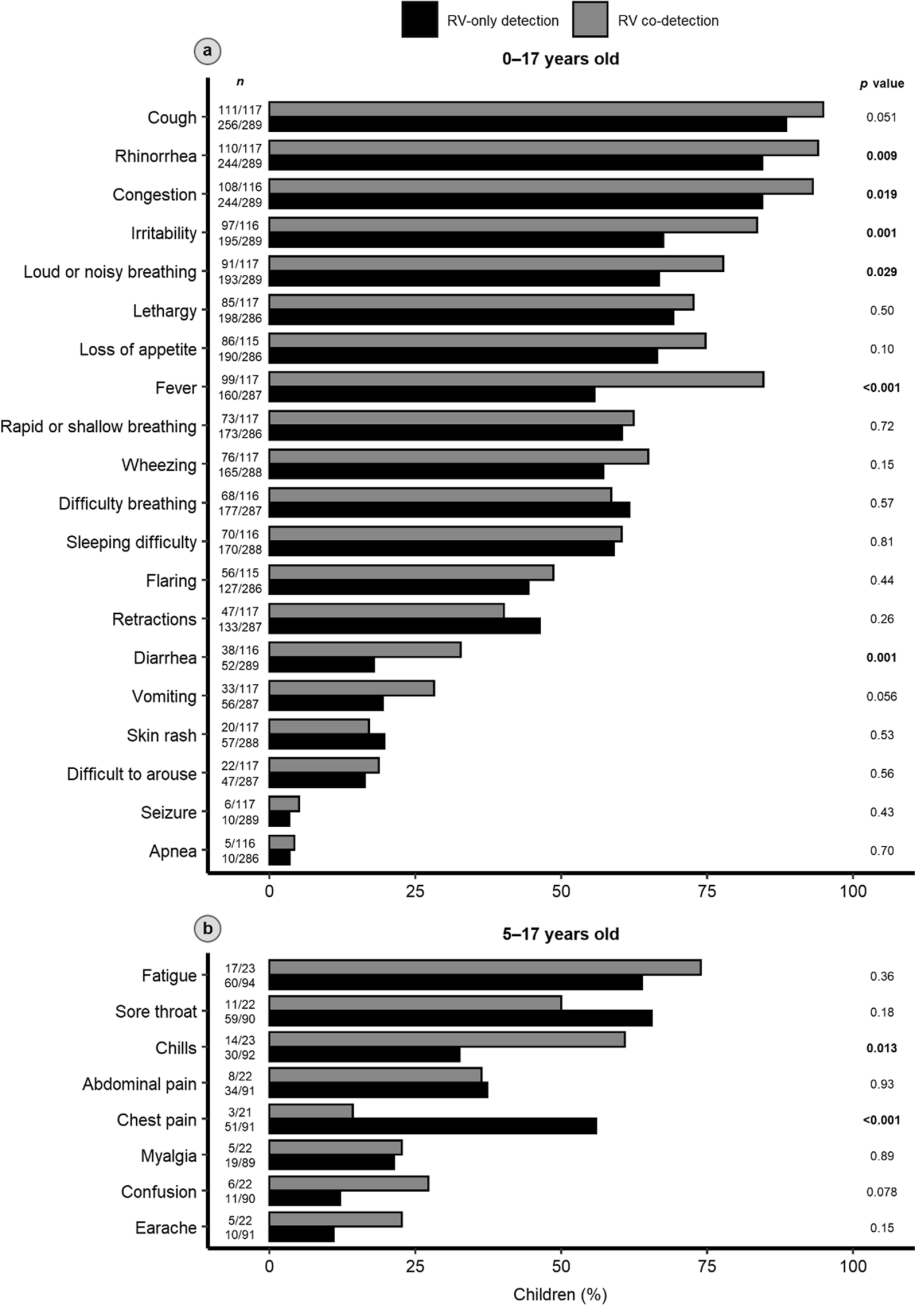
**a** Signs and symptoms of acute respiratory infection in 406 children with rhinovirus (RV)-only detection or RV co-detection in Middle Tennessee. **b** Signs and symptoms specific to children ≥ 5 years old are presented separately. *p* values were calculated using Pearson’s *χ*^2^ test

The most common presenting signs and symptoms were cough (*n* = 367; 90.4%), rhinorrhea (*n* = 354; 87.2%), and congestion (*n* = 352; 86.7%). Children with RV co-detection were more likely than children with RV-only detection to present with rhinorrhea, congestion, irritability, loud or noisy breathing, fever, diarrhea, and chills (Fig. 2). The subset of children with RV/RSV co-detection had a distinct clinical presentation; they were more likely than children with RV-only detection to present with cough, rhinorrhea, congestion, irritability, loud or noisy breathing, rapid or shallow breathing, fever, difficulty breathing, wheezing, nasal flaring, retractions, diarrhea, and apnea (Additional file 1: Fig. S1).

### Diagnoses of children with RV-only detection and RV co-detection

**Fig. 3:**
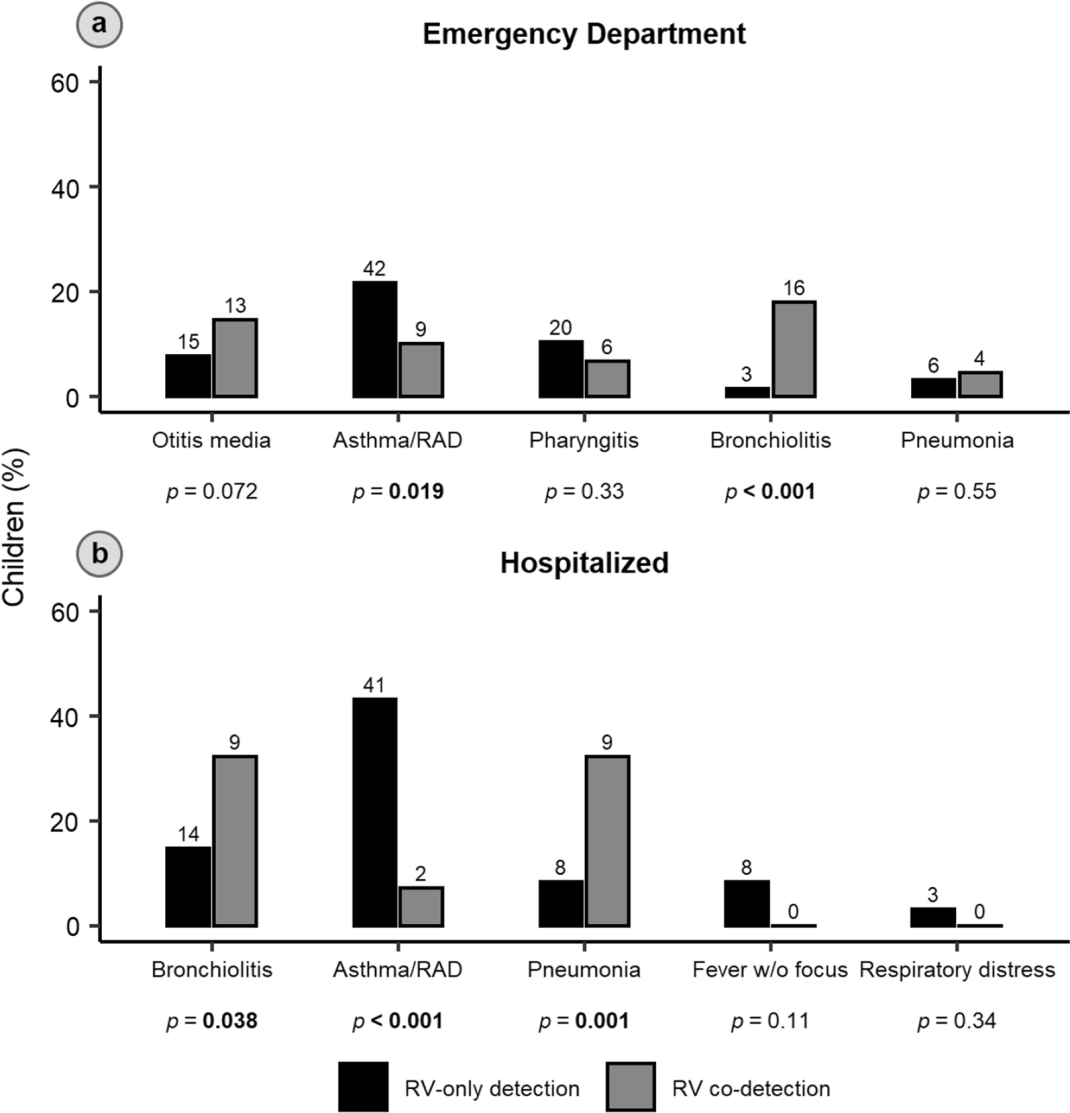
Most common diagnoses in children with single or co-detected rhinovirus-associated acute respiratory infection in Middle Tennessee (**a**) discharged from the emergency department or (**b**) hospitalized. *p* values were calculated using Pearson’s *χ*^2^ test. *RAD* reactive airway disease, *RV* rhinovirus

Of all 406 children with RV, 283 (69.7%) were discharged from the ED and 123 (30.3%) were hospitalized. The diagnoses of the study population, stratified by RV detection status, are summarized in Fig. 3 and Additional file 1: Fig. S2. The most common diagnoses in children discharged from the ED were asthma (*n* = 51; 18.0%), otitis media (*n* = 28; 9.9%), and pharyngitis (*n* = 26; 9.2%). Children with RV co-detection who were discharged from the ED were less likely to be diagnosed with asthma or reactive airway disease (RAD) than children with RV-only detection (10.1% vs. 21.6%, respectively; *p* = 0.019) but more likely to be diagnosed with bronchiolitis (18.0% vs. 1.5%, respectively; *p* < 0.001). Though children with RV/RSV co-detection were also more likely to be diagnosed with bronchiolitis than children with RV-only detection (35.0% vs. 1.5%; *p* < 0.001), the likelihood of an asthma/RAD diagnosis was similar between the two groups (15.0% vs. 21.6%, respectively; *p* = 0.49).

The most common diagnoses in hospitalized children were asthma/RAD (*n* = 43; 35.0%), bronchiolitis (*n* = 23; 18.7%), and pneumonia (*n* = 17; 13.8%). Hospitalized children with RV-codetection were less likely to be diagnosed with asthma/RAD than children with RV-only detection (7.1% vs. 43.2%, respectively; *p* < 0.001) but more likely to be diagnosed with bronchiolitis (32.1% vs. 14.7%, respectively; *p* = 0.038) and pneumonia (32.1% vs. 8.4%, respectively; *p* = 0.001). Similarly, children with RV/RSV co-detection were less likely to be diagnosed with asthma/RAD than children with RV-only detection (0.0% vs. 43.2%, respectively; *p* < 0.001) but more likely to be diagnosed with bronchiolitis (56.2% vs. 14.7%, respectively; *p* < 0.001) and pneumonia (32.1% vs. 8.4%, respectively; *p* = 0.048).

### Outcomes of children with RV-only detection and RV co-detection

The proportions of children who were hospitalized did not significantly differ between the RV-only detection (32.9%) and RV co-detection (23.9%) groups (*p* = 0.076; Table 1). Among children who were hospitalized, the proportions of children who were admitted to the ICU (9.5% and 14.3%, respectively; *p* = 0.47) or required supplemental oxygen (38.9% and 57.1%, respectively; *p* = 0.088) did not differ. In addition, the mean lengths of stay were not different between groups (2.0 ± 2.6 days and 2.9 ± 2.2 days, respectively; *p* = 0.097). Two children (both of whom had RV-only detection) were intubated, and none of the children received extracorporeal membrane oxygenation or died. Results from our logistic regression model for odds of hospitalization are shown in Table 2. We found that age at enrollment (aOR [95% CI], 0.86 [0.79–0.93]; *p* < 0.001), black, non-Hispanic origin (0.26 [0.14–0.45]; *p* < 0.001), Hispanic origin (0.34 [0.18–0.63]; *p* < 0.001), and history of asthma (3.37 [1.82–6.40]; *p* < 0.001) were associated with odds of hospitalization. Importantly, RV co-detection was not a significant predictor of hospitalization (0.59 [0.34–1.00], *p* = 0.054). Our subgroup analysis comparing children with RV-only detection and those with RV/RSV co-detection (Table 3) showed a similar pattern of results, with one exception; the odds of hospitalization were lower in boys than in girls (0.56 [0.34–0.92]; *p* = 0.023). RV/RSV co-detection was not a significant predictor of hospitalization (1.23 [0.56–2.67], *p* = 0.60).

**Table 2:** Multivariable logistic regression model of hospitalization in 405 children with RV-only detection (*n* = 289) or RV co-detection (*n* = 116)

Predictors of hospitalization	aOR (95% CI)	*p* value
RV co-detection	0.59 (0.33–1.03)	0.063
Age at enrollment in years	0.86 (0.80–0.93)	**< 0.001**
Male	0.64 (0.40–1.02)	0.060
Race and Hispanic origin		
Non-Hispanic white	Referent	Referent
Non-Hispanic black	0.26 (0.15–0.46)	**< 0.001**
Non-Hispanic other	1.25 (0.56–2.82)	0.58
Hispanic	0.34 (0.18–0.64)	**< 0.001**
Tobacco smoke exposure^a^	0.92 (0.57–1.48)	0.72
History of asthma	3.37 (1.80–6.32)	**< 0.001**

Values in bold indicate *p* < 0.05

^a^One record with missing data on tobacco smoke exposure was dropped from the model

**Table 3:** Multivariable logistic regression model of hospitalization in 324 children with RV-only detection (*n* = 289) or RV/RSV co-detection (*n* = 35)

Predictors of hospitalization	aOR (95% CI)	*p* value
RV/RSV co-detection	1.23 (0.55–2.74)	0.61
Age at enrollment in years	0.83 (0.76–0.91)	**< 0.001**
Male	0.56 (0.34–0.93)	**0.024**
Race and Hispanic origin		
Non-Hispanic white	Referent	Referent
Non-Hispanic black	0.27 (0.14–0.51)	**< 0.001**
Non-Hispanic other	0.99 (0.40–2.44)	0.99
Hispanic	0.34 (0.17–0.69)	**0.003**
Tobacco smoke exposure^a^	0.87 (0.52–1.46)	0.60
History of asthma	3.96 (1.93–8.13)	**< 0.001**

Values in bold indicate *p* < 0.05

^a^One record with missing data on tobacco smoke exposure was dropped from the model

### Analysis of children < 2 years old with RV/RSV co-detection, RV-only detection, or RSV-only detection

The clinical characteristics and outcomes of children with RV/RSV co-detection and those with RSV-only detection were largely similar, while those of children with RV/RSV co-detection and those with RV-only detection were largely distinct (Additional file 1: Table S2). Notably, young children with RV/RSV co-detection were more likely than young children with RV-only detection to be admitted to the ICU (21.4% vs. 4.3%, respectively; *p* = 0.040) and require supplemental oxygen (64.3% vs. 27.7%, respectively; *p* = 0.012).

### Seasonality

**Fig. 4:**
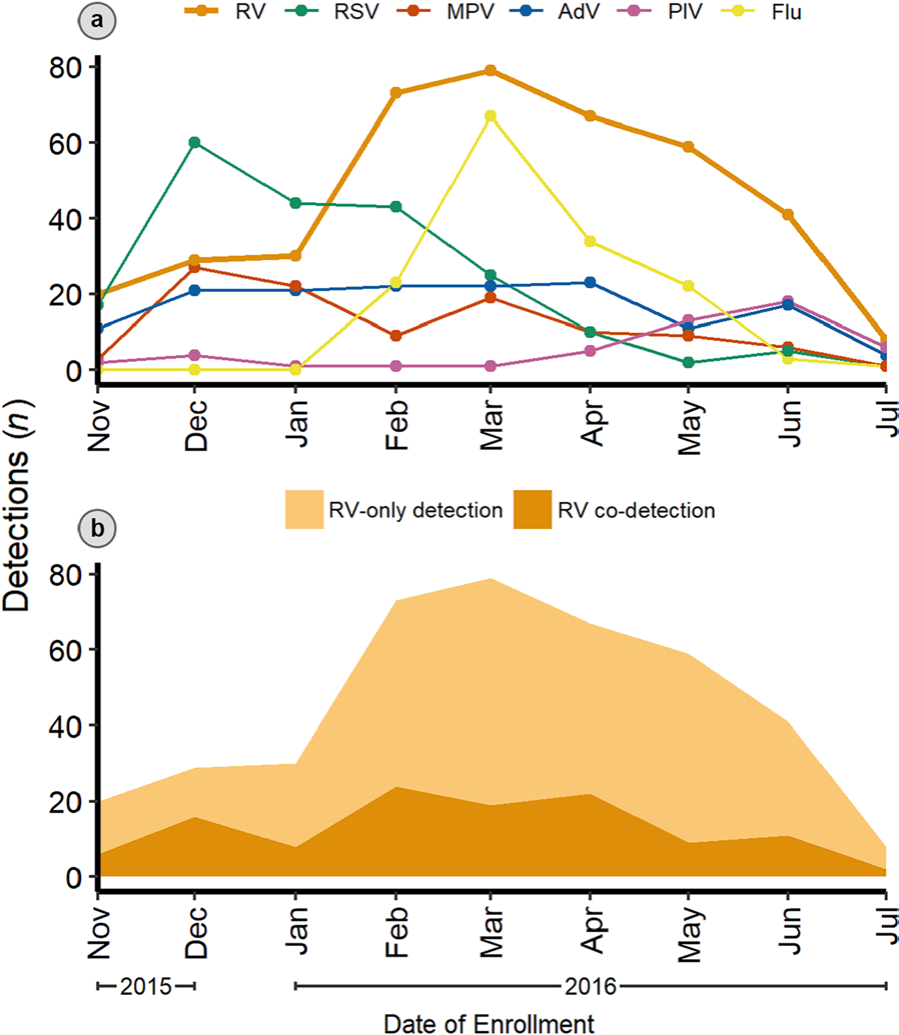
**a** Seasonality of rhinovirus (RV), respiratory syncytial virus (RSV), metapneumovirus (MPV), adenovirus (AdV), parainfluenza virus (PIV), and influenza (Flu) detected in 904 virus-positive children with acute respiratory infection in Middle Tennessee between November 15, 2015, and July 15, 2016. **b** Area plot of RV-only detections and RV co-detections in the same population and period

RV was the most common virus throughout the study months except in early winter (December) and midwinter (January), when RSV predominated (Fig. 4). Overall, RV detections peaked in early spring (March) and dropped to a nadir in midsummer (July). RV-only detections peaked in early spring (March), while RV co-detections peaked in late winter (February).

## Discussion

In our prospective viral surveillance study of 1250 children with ARI enrolled from November 2015 to July 2016, we found that RV was the most frequently detected respiratory virus, while RV/RSV was the most frequent virus co-detection. We also found that children with RV co-detection were younger, on average, than children with RV-only detection, had a longer duration of illness at presentation, were similarly or more likely to present with all but one of the symptoms reported, were less likely to be diagnosed with asthma/RAD but more likely to be diagnosed with bronchiolitis if discharged from the ED, and were less likely to be diagnosed with asthma/RAD but more likely to be diagnosed with bronchiolitis or pneumonia if hospitalized. However, RV co-detection was not significantly associated with hospitalization in unadjusted or adjusted analyses, and among children who were hospitalized, the proportions of those who were admitted to the ICU or required supplemental oxygen were similar between groups, as were the lengths of stay. Finally, we performed a subgroup analysis of children with RV-only detection compared with those with RV/RSV co-detection and found that, although outcomes did not significantly differ between groups, the clinical presentation of children with RV/RSV co-detection was distinct from that of children with any RV co-detection.

The RV/RSV pair was the most frequent co-detection in our study, which is expected because RV and RSV are the most common causes of upper and lower respiratory infection, respectively, in children and the two most common respiratory viruses in viral surveillance studies [16, 17]. Given that RSV was positive in more than one-third of co-detected cases, the clinical characteristics of RSV-associated ARI may explain many of the differences between children with RV-only detection and those with RV co-detection. For example, RSV is the leading cause of hospitalization in children with ARI, which may explain our finding that children with RV co-detection were younger, on average, than those with RV-only detection [18]. In support, we showed that most children with RSV were < 2 years old, and in our subgroup analysis comparing children with RV-only detection and those with RV/RSV co-detection, the difference in the age at enrollment was more pronounced than in the main analysis.

The effect of viral co-infection on the severity of ARI remains unclear, and results from systematic reviews and meta-analyses are conflicting [7, 8, 19–21]. The heterogeneity of results may be explained by distinct virus-virus interactions that are obscured by analyses of aggregated data. DaPalma et al. identified 15 subtypes of virus-virus interactions that may ultimately attenuate or accentuate the severity of clinical disease [22]. In our subgroup analyses, we compared children with RV-only detection and those with RV/RSV co-detection and found that co-detection was not associated with hospitalization. In children who were hospitalized, we found no evidence that RV/RSV co-detection conferred worse outcomes. In support, Li et al. conducted a systematic review and meta-analysis comparing children < 5 years old with RSV-only detection and those with RSV co-detection and found no evidence that RV/RSV co-detection portended worse outcomes [23]. However, Comte et al. found that children ≤ 2 years old with RV/RSV co-detection were more likely than children with RV-only detection to have severe disease [24]. The discrepancy between our results and those of Comte et al. is likely a result of age differences; RV is associated with a considerable proportion of asthma exacerbations, and most asthma cases are diagnosed in children > 2 years old—a population that was not included in their study [24, 25]. Indeed, asthma/RAD was the most common diagnosis in our study, and most children with asthma/RAD were 2–17 years old. Our main analysis also showed no differences in outcome between children with RV-only and those with RV co-detection, but further studies of specific RV/non-RV pairs are needed to validate these results.

Based on the differential clinical characteristics of children with RV-only detection and those with RV/RSV co-detection as well as the results reported by Comte et al., we speculated that RSV was driving the clinical presentation of children with RV/RSV co-detection [24]. Compared to children with RV-only detection, those with RV/RSV co-detection were more likely to have multiple signs and symptoms associated with lower respiratory tract infections, and they were more likely to be diagnosed with bronchiolitis if discharged from the ED and more likely to be diagnosed with bronchiolitis or pneumonia if admitted. To test our hypothesis, we performed a comparison of the clinical characteristics and outcomes of children < 2 years old with RV/RSV co-detection and those with RV-only or RSV-only detection. We found that the clinical characteristics and outcomes of children < 2 years old with RV/RSV co-detection and those with RSV-only detection were alike, while children < 2 years old with RV-only detection were less likely than those with RV/RSV co-detection to have signs and symptoms of respiratory distress, less likely to be diagnosed with bronchiolitis if discharged from the ED or admitted, and less likely to be admitted to the ICU and require supplemental oxygen if hospitalized. Therefore, our results are consistent with the hypothesis that RV acts as a “bystander” in young children with RV/RSV co-detection [26]. Nevertheless, our study lacks long-term follow-up, precluding us from studying whether RV/RSV co-detection predisposes children to subsequent wheezing in later life, which must be addressed in a future study.

The strengths of our study include enrollment of children of all ages from both the ED and inpatient service, systematic collection of nose and/or throat swabs from each participant regardless of provider-ordered testing, and RT-qPCR testing for a wide spectrum of common viral etiologies of pediatric ARI. We also note some limitations. First, our PCR panel did not include all frequently encountered respiratory viruses, such as bocavirus and endemic coronaviruses. We nonetheless detected at least one virus in 72.3% of all children, which is equivalent to the proportion of children with ARI in a previous study who tested positive for at least one virus using a pathogen panel that included bocavirus and the endemic coronaviruses [27]. Second, the catchment area in our single-center study included nine counties in Middle Tennessee, and the dates of enrollment spanned less than a year; therefore, our results may not be generalizable to other regions in the United States. In addition, as with many other viruses, pathogenicity is affected by the specific strain or genotype of RV, which is subject to seasonal variation [28]. Therefore, that our study spans a single respiratory season is an important limitation, and future studies should span multiple seasons. Third, we interpreted any Ct value (up to 45, the number of assay cycles) to indicate pathogen presence. While very low viral loads can be difficult to resolve from nonspecific amplification, all PCR growth curves were inspected for features of authentic target amplification, followed by retesting of specimens yielding ambiguous results. Furthermore, of 1,072 detections, only 0.4% (*n* = 4) were defined as positive based on a Ct value exceeding 40. Finally, we performed a subgroup analysis of only the most common RV/non-RV pair, namely RV/RSV, because of sample size limitations. The clinical significance of other RV/non-RV pairs should be explored in further studies, as should the clinical significance of individual RV species and serotypes paired with RSV or other respiratory viruses. In addition, the clinical significance of these pairs should be explored in the context of specific clinical diagnoses, such as asthma exacerbation, bronchiolitis, and pneumonia.

In conclusion, aggregated analysis showed no evidence that RV co-detection was associated with poorer outcomes, consistent with previous reports. However, our subset analyses showed that the clinical significance of RV co-detection is heterogeneous and varies by virus pair and age group. Future studies of RV co-detection should incorporate analyses of RV/non-RV pairs and include age as a key covariate of RV contribution to clinical manifestations and infection outcomes.

## Supplementary Information

**Additional file 1**. Supplementary analyses of the clinical characteristics and outcomes of children with single or co-detected rhinovirus-associated acute respiratory infection in Middle Tennessee.

## Data Availability

The data that support the findings of this study are available from the corresponding author on reasonable request.

## Abbreviations

ARI: Acute respiratory infection
RV: Rhinovirus
ED: Emergency department
ICU: Intensive care unit
RT-qPCR: Reverse transcription quantitative polymerase chain reaction
RSV: Respiratory syncytial virus
AdV: Adenovirus
Flu: Influenza
MPV: Metapneumovirus
PIV: Parainfluenza virus
Ct: Cycle threshold
SD: Standard deviation
IQR: Interquartile range
aOR: Adjusted odds ratio
CI: Confidence interval
RAD: Reactive airway disease
