# Supplementary material for: Clinical characteristics and outcomes of children with single or co-detected rhinovirus-associated acute respiratory infection in Middle Tennessee
Source: BMC Infect Dis. 2023 Mar 7;23:136. doi: 10.1186/s12879-023-08084-4 (PMC9990557; doi:10.1186/s12879-023-08084-4)

**Table S1.** Clinical characteristics and outcomes of 904 children in Middle Tennessee with at least one viral detection, stratified by RV detection status.

|  | **RV-positive (*n*=406)** | **RV-negative (*n*=498)** | ***p* value*** |
| --- | --- | --- | --- |
| **Clinical characteristics** |  |  |  |
| Age at enrollment in years |  |  |  |
| Mean (SD) | 3.8 (4.1) | 3.2 (3.4) | **0.025** |
| Median (IQR) | 2.3 (0.8–5.8) | 1.9 (0.9–4.5) |  |
| Male—*n* (%) | 247 (60.8) | 239 (48.0) | **<0.001** |
| Race and Hispanic origin—*n* (%) |  |  | 0.35 |
| Hispanic | 97 (23.9) | 131 (26.3) |  |
| Non-Hispanic white | 123 (30.3) | 158 (31.7) |  |
| Non-Hispanic black | 158 (38.9) | 187 (37.6) |  |
| Non-Hispanic other | 28 (6.9) | 22 (4.4) |  |
| Illness duration at presentation in days |  |  |  |
| Mean (SD) | 3.6 (2.3) | 4.0 (2.3) | **0.004** |
| Median (IQR) | 3 (2–5) | 3 (2–5) |  |
| Breastfeeding history^a^—*n* (%) | 135/189 (71.4) | 178/253 (70.4) | 0.81 |
| Premature birth^a^—*n* (%) | 36/185 (19.5) | 61/252 (24.2) | 0.24 |
| Day care or preschool attendance^b^—*n* (%) | 90/284 (31.7) | 131/391 (33.5) | 0.62 |
| School attendance^c^—*n* (%) | 109/117 (93.2) | 100/107 (93.5) | 0.93 |
| Smoke exposure—*n* (%) | 158/405 (39.0) | 159 (31.9) | **0.027** |
| Gestational smoke exposure^a^—*n* (%) | 59/401 (14.7) | 61 (12.2) | 0.28 |
| Prior antiviral use—*n* (%) | 1/403 (0.2) | 7/494 (1.4) | 0.064 |
| Prior antibiotic use—*n* (%) | 28/403 (6.9) | 70/495 (14.1) | **<0.001** |
| Underlying medical condition—*n* (%) | 215 (53.0) | 213 (42.8) | **0.002** |
| History of asthma—*n* (%) | 114 (53.0) | 70 (32.9) | **<0.001** |
| **Signs and symptoms** |  |  |  |
| Abdominal pain^c^—*n* (%) | 42/113 (37.2) | 47/101 (46.5) | 0.17 |
| Apnea—*n* (%) | 15/402 (3.7) | 26/492 (5.3) | 0.27 |
| Chest pain^c^—*n* (%) | 54/112 (48.2) | 35/101 (34.7) | **0.045** |
| Chills^c^—*n* (%) | 44/115 (38.3) | 64/103 (62.1) | **<0.001** |
| Confusion^c^—*n* (%) | 17/112 (15.2) | 22/105 (21.0) | 0.27 |
| Congestion—*n* (%) | 352/405 (86.9) | 421/497 (84.7) | 0.35 |
| Cough—*n* (%) | 367 (90.4) | 450 (90.4) | 0.99 |
| Diarrhea—*n* (%) | 90/405 (22.2) | 137 (27.5) | 0.069 |
| Difficult to arouse—*n* (%) | 69/404 (17.1) | 102 (20.5) | 0.19 |
| Difficulty breathing—*n* (%) | 245/403 (60.8) | 229/495 (46.3) | **<0.001** |
| Earache^c^—*n* (%) | 15/113 (13.3) | 24/103 (23.3) | 0.056 |
| Fatigue^c^—*n* (%) | 77/117 (65.8) | 75/105 (71.4) | 0.37 |
| Fever—*n* (%) | 259/404 (64.1) | 424/494 (85.8) | **<0.001** |
| Irritability—*n* (%) | 292/405 (72.1) | 401/496 (80.8) | **0.002** |
| Lethargy—*n* (%) | 283/403 (70.2) | 397/495 (80.2) | **<0.001** |
| Loss of appetite—*n* (%) | 276/401 (68.8) | 389/492 (79.1) | **<0.001** |
| Loud or noisy breathing—*n* (%) | 284 (70.0) | 323 (64.9) | 0.10 |
| Myalgia^c^—*n* (%) | 24/111 (21.6) | 41/102 (40.2) | **0.003** |
| Nasal flaring—*n* (%) | 183/401 (45.6) | 192/493 (38.9) | **0.044** |
| Rapid or shallow breathing—*n* (%) | 246/403 (61.0) | 267/497 (53.7) | **0.027** |
| Retractions—*n* (%) | 180/404 (44.6) | 165/485 (34.0) | **0.001** |
| Rhinorrhea—*n* (%) | 354 (87.2) | 434/497 (87.3) | 0.95 |
| Seizure—*n* (%) | 16 (3.9) | 13/495 (2.6) | 0.27 |
| Skin rash—*n* (%) | 77/405 (19.0) | 83 (16.7) | 0.36 |
| Sleeping difficulty—*n* (%) | 240/404 (59.4) | 313/497 (63.0) | 0.27 |
| Sore throat^c^—*n* (%) | 70/112 (62.5) | 61/101 (60.4) | 0.75 |
| Vomiting—*n* (%) | 89/404 (22.0) | 134/497 (27.0) | 0.088 |
| Wheezing—*n* (%) | 241/405 (59.5) | 256/497 (51.5) | **0.016** |
| **Final diagnoses** |  |  |  |
| Discharged from ED |  |  |  |
| Asthma/RAD—*n* (%) | 51/283 (18.0) | 31/397 (7.8) | **<0.001** |
| Bronchiolitis—*n* (%) | 19/283 (6.7) | 44/397 (11.1) | 0.053 |
| Otitis media—*n* (%) | 28/283 (9.9) | 56/397 (14.1) | 0.10 |
| Pharyngitis—*n* (%) | 26/283 (9.2) | 38/397 (9.6) | 0.87 |
| Pneumonia—*n* (%) | 10/283 (3.5) | 9/397 (2.3) | 0.32 |
| Hospitalized |  |  |  |
| Asthma/RAD—*n* (%) | 43/123 (35.0) | 11/101 (10.9) | **<0.001** |
| Bronchiolitis—*n* (%) | 23/123 (18.7) | 39/101 (38.6) | **<0.001** |
| Fever without focus—*n* (%) | 8/123 (6.5) | 5/101 (5.0) | 0.62 |
| Pneumonia—*n* (%) | 17/123 (13.8) | 18/101 (17.8) | 0.41 |
| Respiratory distress—*n* (%) | 3/123 (2.4) | 6/101 (5.9) | 0.18 |
| **Outcomes** |  |  |  |
| Hospitalized—*n* (%) | 123 (30.3) | 101 (20.3) | **<0.001** |
| ICU admission—*n* (%) | 13 (10.6) | 12 (11.9) | 0.76 |
| Supplemental oxygen use—*n* (%) | 53 (43.1) | 53 (52.5) | 0.16 |
| Length of stay in days |  |  |  |
| Mean (SD) | 2.2 (2.5) | 2.8 (3.3) | 0.18 |
| Median (IQR) | 1.0 (1–2) | 2 (1–3) |  |

**p* values were calculated using Pearson’s *χ*^2^ test for categorical variables and the two-sample *t*-test with unequal variances for continuous variables. ^a^Children <2 years old. ^b^Children <5 years old. ^c^Children ≥5 years old.

**Abbreviations:** RV, rhinovirus; SD, standard deviation; IQR, interquartile range; ED, emergency department; RAD, reactive airway disease; ICU, intensive care unit.

**Table S2.** Clinical characteristics and outcomes of 252 children <2 years old with RV/RSV co-detection, RV-only detection, or RSV-only detection in Middle Tennessee.

|  | **RV/RSV co-detection (*n*=28)** | **RV-only detection (*n*=123)** | ***p* value*** | **RSV-only detection (*n*=101)** | ***p* value*** |
| --- | --- | --- | --- | --- | --- |
| **Clinical characteristics** |  |  |  |  |  |
| Age at enrollment in months |  |  |  |  |  |
| Mean (SD) | 7.4 (6.7) | 8.7 (7.3) | 0.37 | 8.7 (6.9) | 0.38 |
| Median (IQR) | 4.3 (2.1–12.2) | 6.2 (2.0–13.6) |  | 6.4 (2.9–13.7) |  |
| Male—*n* (%) | 15 (53.6) | 75 (61.0) | 0.47 | 51 (50.5) | 0.77 |
| Race and Hispanic origin—*n* (%) |  |  | 0.074 |  | 0.051 |
| Hispanic | 5 (17.9) | 38 (30.9) |  | 21 (20.8) |  |
| Non-Hispanic white | 10 (35.7) | 48 (39.0) |  | 48 (47.5) |  |
| Non-Hispanic black | 7 (25.0) | 29 (23.6) |  | 27 (26.7) |  |
| Non-Hispanic other | 6 (21.4) | 8 (6.5) |  | 5 (5.0) |  |
| Illness duration at presentation in days |  |  |  |  |  |
| Mean (SD) | 5.2 (3.2) | 3.5 (2.3) | **0.006** | 4.1 (1.8) | 0.066 |
| Median (IQR) | 5 (3–7) | 3 (2–5) |  | 4 (3–5) |  |
| Breastfeeding history^a^—*n* (%) | 19 (67.9) | 90 (73.2) | 0.57 | 78 (77.2) | 0.31 |
| Premature birth^a^—*n* (%) | 6/26 (23.1) | 22/121 (18.2) | 0.56 | 24/100 (24.0) | 0.92 |
| Day care or preschool attendance^b^—*n* (%) | 8 (28.6) | 20/119 (16.8) | 0.15 | 26 (25.7) | 0.76 |
| Smoke exposure—*n* (%) | 15 (53.6) | 49 (39.8) | 0.18 | 32 (31.7) | **0.033** |
| Gestational smoke exposure^a^—*n* (%) | 7 (25.0) | 21 (17.1) | 0.33 | 12 (11.9) | 0.083 |
| Prior antiviral use—*n* (%) | 0 (0.0) | 0 (0.0) | NA | 1/100 (1.0) | 0.60 |
| Prior antibiotic use—*n* (%) | 3 (10.7) | 6/122 (4.9) | 0.24 | 16/100 (16.0) | 0.49 |
| Underlying medical condition—*n* (%) | 10 (35.7) | 34 (27.6) | 0.40 | 30 (29.7) | 0.54 |
| History of asthma—*n* (%) | 0/10 (0.0) | 8/34 (23.5) | 0.090 | 5/30 (16.7) | 0.17 |
| **Signs and symptoms** |  |  |  |  |  |
| Apnea—*n* (%) | 4/27 (14.8) | 4/122 (3.3) | **0.016** | 11/99 (11.1) | 0.60 |
| Congestion—*n* (%) | 28 (100.0) | 105 (85.4) | **0.031** | 95/100 (95.0) | 0.23 |
| Cough—*n* (%) | 28 (100.0) | 104 (84.6) | **0.026** | 99 (98.0) | 0.45 |
| Diarrhea—*n* (%) | 10 (35.7) | 38 (30.9) | 0.62 | 26 (25.7) | 0.30 |
| Difficult to arouse—*n* (%) | 5 (17.9) | 15/121 (12.4) | 0.44 | 17 (16.8) | 0.90 |
| Difficulty breathing—*n* (%) | 23 (82.1) | 66/122 (54.1) | **0.006** | 71 (70.3) | 0.21 |
| Fever—*n* (%) | 24 (85.7) | 84/122 (68.9) | 0.073 | 74 (73.3) | 0.17 |
| Irritability—*n* (%) | 25 (89.3) | 98 (79.7) | 0.24 | 91/100 (91.0) | 0.78 |
| Lethargy—*n* (%) | 22 (78.6) | 85/121 (70.2) | 0.38 | 74/100 (74.0) | 0.62 |
| Loss of appetite—*n* (%) | 22 (78.6) | 86/122 (70.5) | 0.39 | 79 (78.2) | 0.97 |
| Loud or noisy breathing—*n* (%) | 24 (85.7) | 80 (65.0) | **0.033** | 80 (79.2) | 0.44 |
| Nasal flaring—*n* (%) | 21 (75.0) | 57/121 (47.1) | **0.008** | 53 (52.5) | **0.033** |
| Rapid or shallow breathing—*n* (%) | 25 (89.3) | 65/122 (53.3) | **<0.001** | 71 (70.3) | **0.042** |
| Retractions—*n* (%) | 18 (64.3) | 47/122 (38.5) | **0.013** | 53/100 (53.0) | 0.29 |
| Rhinorrhea—*n* (%) | 27 (96.4) | 105 (85.4) | 0.11 | 90/100 (90.0) | 0.28 |
| Seizure—*n* (%) | 2 (7.1) | 6 (4.9) | 0.63 | 0 (0.0) | **0.007** |
| Skin rash—*n* (%) | 6 (21.4) | 32/122 (26.2) | 0.60 | 15 (14.9) | 0.40 |
| Sleeping difficulty—*n* (%) | 20 (71.4) | 74 (60.2) | 0.27 | 78/100 (78.0) | 0.47 |
| Vomiting—*n* (%) | 10 (35.7) | 33/122 (27.0) | 0.36 | 30 (29.7) | 0.54 |
| Wheezing—*n* (%) | 23 (82.1) | 55/122 (45.1) | **<0.001** | 74 (73.3) | 0.34 |
| **Final diagnoses** |  |  |  |  |  |
| Discharged from ED |  |  |  |  |  |
| Asthma/RAD—*n* (%) | 1/14 (7.1) | 2/76 (2.6) | 0.39 | 5/65 (7.7) | 0.94 |
| Bronchiolitis—*n* (%) | 7/14 (50.0) | 3/76 (3.9) | **<0.001** | 28/65 (43.1) | 0.64 |
| Hospitalized |  |  |  |  |  |
| Asthma/RAD—*n* (%) | 0/14 (0.0) | 2/47 (4.3) | 0.43 | 2/36 (5.6) | 0.37 |
| Bronchiolitis—*n* (%) | 9/14 (64.3) | 14/47 (29.8) | **0.019** | 24/36 (66.7) | 0.87 |
| **Outcomes** |  |  |  |  |  |
| Hospitalized—*n* (%) | 14 (50.0) | 47 (38.2) | 0.25 | 36 (35.6) | 0.17 |
| ICU admission—*n* (%) | 3 (21.4) | 2 (4.3) | **0.040** | 6 (16.7) | 0.69 |
| Supplemental oxygen use—*n* (%) | 9 (64.3) | 13 (27.7) | **0.012** | 21 (58.3) | 0.70 |
| Length of stay in days |  |  |  |  |  |
| Mean (SD) | 3.2 (2.5) | 2.4 (3.0) | 0.34 | 2.6 (2.9) | 0.49 |
| Median (IQR) | 2 (2–3.75) | 2 (1–2) |  | 2 (1–3) |  |

**p* values were calculated using Pearson’s *χ*^2^ test for categorical variables and the two-sample *t*-test with unequal variances for continuous variables. ^a^Children <2 years old. ^b^Children <5 years old. ^c^Children ≥5 years old.

**Abbreviations:** RV, rhinovirus; RSV, respiratory syncytial virus; SD, standard deviation; IQR, interquartile range; ED, emergency department; RAD, reactive airway disease; ICU, intensive care unit.

**Figure S1.** Signs and symptoms of acute respiratory infection in 325 children with rhinovirus (RV)-only detection or RV co-detected with respiratory syncytial virus (RSV) in Middle Tennessee. *p* values were calculated using Pearson’s *χ*^2^ test.


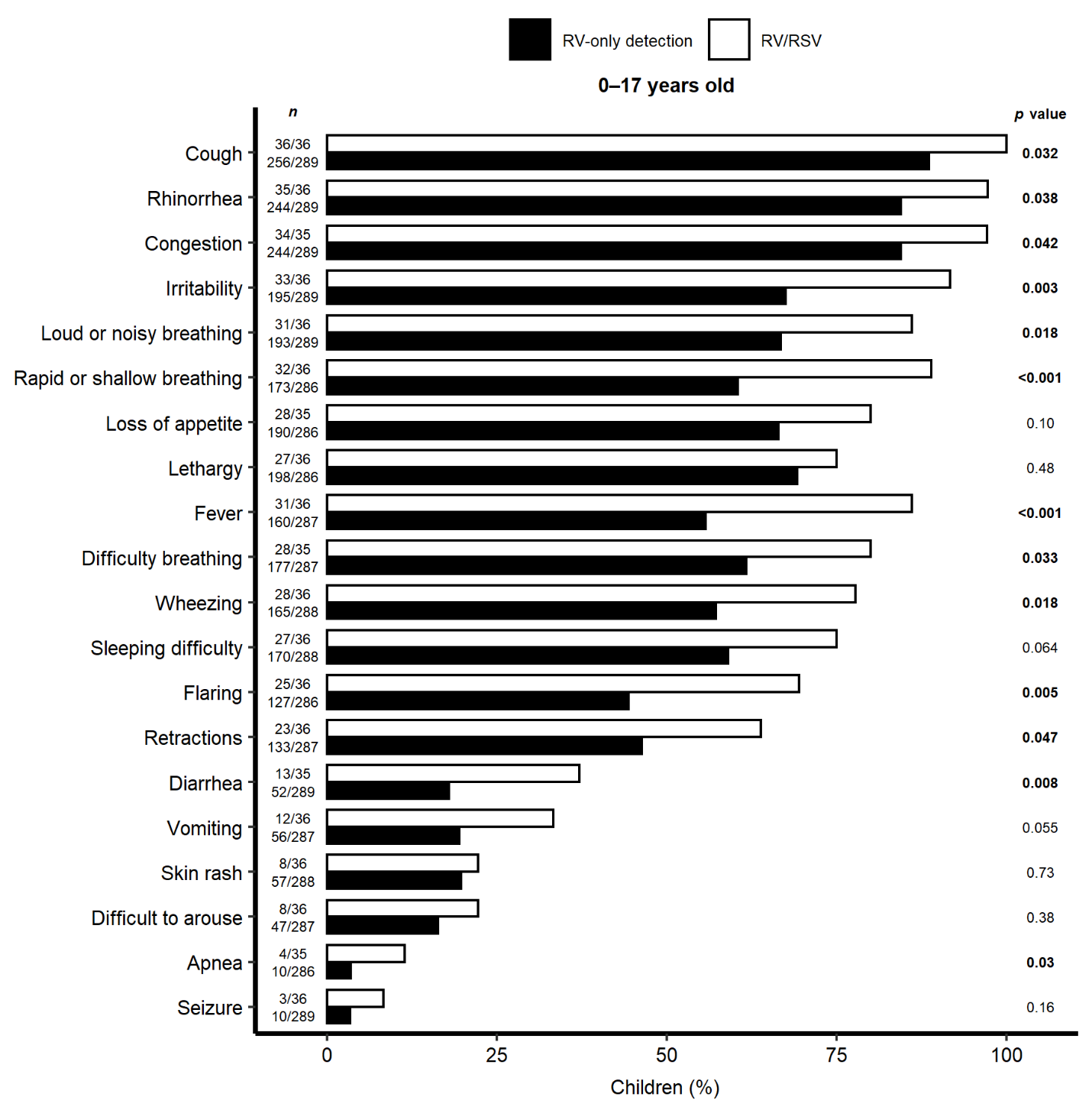


**Figure S2.** Most common diagnoses in children with acute respiratory infection associated with rhinovirus-only detection or rhinovirus co-detected with respiratory syncytial virus in Middle Tennessee **(a)** discharged from the emergency department or **(b)** hospitalized. *p* values were calculated using Pearson’s *χ*^2^ test. **Abbreviations:** RAD, reactive airway disease; RV, rhinovirus; RSV, respiratory syncytial virus.


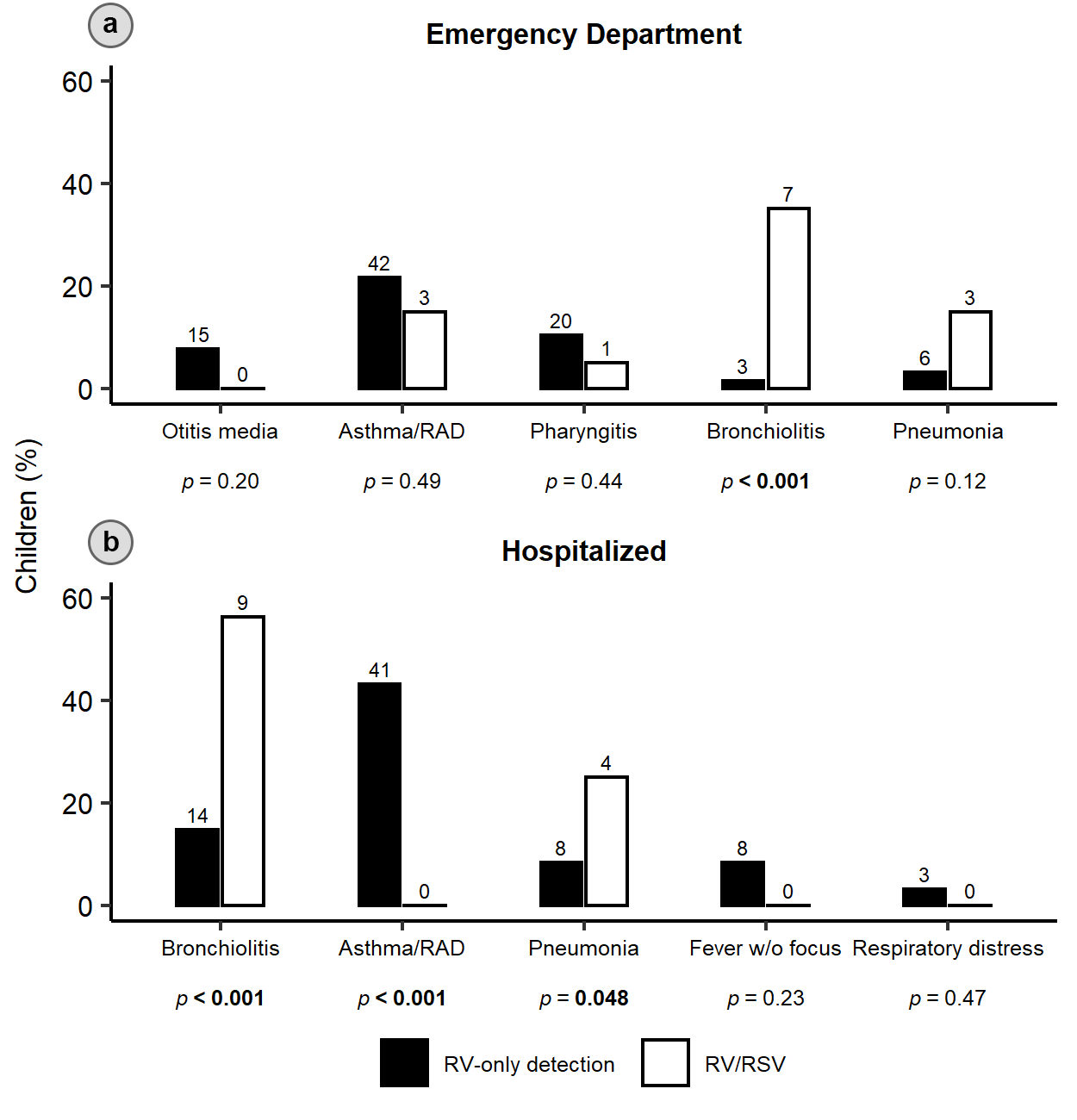

Supplement: Supplementary file 1 — Additional file 1. Supplementary analyses of the clinical characteristics and outcomes of children with single or co-detected rhinovirus-associated acute respiratory infection in Middle Tennessee. [file 12879_2023_8084_MOESM1_ESM.docx]
